# Marital Quality and Mental Health Among Caregiving Dyads

**DOI:** 10.1093/geroni/igaa057.1943

**Published:** 2020-12-16

**Authors:** Christine Proulx, Hanamori Skoblow, Sae Hwang Han

**Affiliations:** 1 University of Missouri, Columbia, Missouri, United States; 2 University of Texas at Austin, Austin, Texas, United States

## Abstract

We examined whether the associations between marital quality and mental health were equally strong among dyads in which one spouse was providing care to a spouse (n = 155), parent (n = 61), or another adult (n = 176). Using Wave 2 of the NSHAP and actor-partner interdependence (APIM) models, we found significant differences (p=.004) among groups. Marital quality was negatively associated with one’s own depressive symptoms (b=-1.29) for husbands in the spousal caregiver group, whereas marital quality was negatively associated with one’s own depressive symptoms for wives in both the parental (b=-1.27) and other adult (b=-1.96) caregiver groups. The only partner effect was the negative association between wives’ marital quality and husbands’ depressive symptoms (b=-2.59) among dyads in which one spouse was a parental caregiver. These results point to the importance of understanding the context of caregiving when examining the protective effect of spouses’ marital quality on mental health.

